# Controlling Nutritional Status (CONUT) Score as a Potential Prognostic Indicator of In-Hospital Mortality, Sepsis and Length of Stay in an Internal Medicine Department

**DOI:** 10.3390/nu15071554

**Published:** 2023-03-23

**Authors:** Nicoletta Miano, Maurizio Di Marco, Salvatore Alaimo, Giuseppe Coppolino, Giuseppe L’Episcopo, Stefano Leggio, Roberto Scicali, Salvatore Piro, Francesco Purrello, Antonino Di Pino

**Affiliations:** Department of Clinical and Experimental Medicine, University of Catania, 95122 Catania, Italy

**Keywords:** malnutrition, CONUT score, in-hospital outcomes, internal medicine

## Abstract

The controlling nutritional status (CONUT) score represents poor nutritional status and has been identified as an indicator of adverse outcomes. Our aim was to evaluate the prognostic role of the CONUT score on in-hospital outcomes in an Internal Medicine Department. This is a retrospective study analyzing data from 369 patients, divided into four groups based on the CONUT score: normal (0–1), mild–high (2–4), moderate–high (5–8), and marked high (9–12). In-hospital all-cause mortality increased from normal to marked high CONUT score group (2.2% vs. 3.6% vs. 13.4% vs. 15.3%, *p* < 0.009). Furthermore, a higher CONUT score was linked to a longer length of hospital stay (LOS) (9.48 ± 6.22 vs. 11.09 ± 7.11 vs. 12.45 ± 7.88 vs. 13.10 ± 8.12, *p* < 0.013) and an increased prevalence of sepsis. The excess risk of a high CONUT score relative to a low CONUT score remained significant after adjusting for confounders (all-cause mortality: OR: 3.3, 95% CI: 1.1–9.7, *p* < 0.02; sepsis: OR: 2.7, 95% CI: 1.5–4.9, *p* < 0.01; LOS: OR: 2.1, 95% CI: 1.2–3.9, *p* < 0.007). The present study demonstrated that an increased CONUT score is related to a higher risk of short-term in-hospital death and complications.

## 1. Introduction

Malnutrition is a disorder caused by insufficient nutrient intake or uptake, which results in altered body composition, decreased physical and cognitive function, and poor clinical outcomes. The condition that results from deficits of macro- and micronutrients, catabolism of protein related to illness and aging referred to as malnutrition, and the states cachexia, sarcopenia, and frailty, are now well established in relation to malnutrition [1,2,3]. Disease-related malnutrition has been estimated to affect 20 to 50% of people worldwide. The risk of malnutrition is higher in older people because of a variety of reasons that frequently affect their nutritional intake [4,5]. Recently, more emphasis has been put on patients’ dietary statuses. Several studies have indeed shown that malnutrition is related to numerous complications during hospitalization, such as infections, bedsores, longer length of stay (LOS), and higher costs related to hospitalization, as compared with patients with a good nutritional status [6,7]. Additionally, nutritional risk has been recognized as an independent predictor of functional status and death rate among institutionalized elderly patients [8]. In line with these considerations, current guidelines recommend that routine clinical evaluation should include the screening of nutritional status, especially among older inpatients. Body mass index (BMI) and albumin serum levels are frequently used for analyzing nutritional status; however, these measures show limitations in clinical use. Currently, no gold standard tool is validated for malnutrition diagnosis and the clinical methods developed to date are not valuable for daily clinical use, mainly because of their effectiveness/cost ratio. The most popular screening tools for identifying malnutrition in patients are nutritional risk screening (NRS), which is considered appropriate for hospital setting, Malnutrition Screening Tool, Patient-generated subjective global assessment and mini nutritional assessment (MNA), which is more suitable for assessing patients in residential settings. However, additional factors, such as anorexia, weight loss over a specific time period, and dietary intake, must also be taken into account [9].

Accordingly, the identification of a screening instrument that could easily identify patients with a higher risk for worse outcomes, particularly in the hospitalized population, remain an unmet clinical need.

The controlling nutritional status (CONUT) score is a screening instrument that permits an objective and simple assessment of the nutritional status of hospitalized patients. It is determined from serum albumin, total cholesterol (TC) serum levels and peripheral lymphocyte count [10], which are parameters often included in routine lab tests at the admission of patients. Moreover, unlike MNA, one of the most used nutritional tools, the CONUT score does not take into account anthropometric measurements, including weight, height, body circumference, and surface, that may be affected by the presence of effusions and edema. Albumin indicates protein reserves, TCs correlate with caloric deficiency, and the lymphocyte count relates to immune defense. A decrement in each element is associated with a higher CONUT score and worse nutritional status. The CONUT score was initially developed to assess acute deterioration in surgical patients, however, it has been demonstrated that a high score has a prognostic role among patients with different clinical conditions, such as malignancies and cardiovascular diseases (ischemic heart disease, stroke and atrial fibrillation) [6,11,12,13]. To date, the CONUT score’s potential predictive value for in-hospital mortality among adult inpatients, in internal medicine departments, has not been investigated. In view of the importance of an early identification of patients with malnutrition, an appropriate nutritional assessment instrument is highly recommended in internal medicine departments. This clinical setting, indeed, involves older, frailer, polymorbid patients who are malnourished or at risk of malnutrition. Furthermore, the decline of nutritional status may be caused by a number of underlying pathological conditions. Nutritional intake can be impaired by vision loss, motor impairments, edentulism, anorexia, dysphagia, and other factors. A well-known etiologic role is also played by other factors, such as the increased prevalence and severity of chronic diseases, polypharmacy, psychological (confusion, sadness, or grief), and social (isolation, loneliness, poverty, or trouble preparing meals) factors [9].

The aim of the present study was to evaluate the CONUT score predictive value for in-hospital mortality, infections and LOS in adult patients hospitalized in an internal medicine department.

## 2. Materials and Methods

### 2.1. Patients

The medical records of patients admitted to the internal medicine department of the Azienda Ospedaliera di Alta Specializzazione Garibaldi Nesima in Catania, Italy, were used to retrospectively collect clinical pathological data, from September to November of the years 2019, 2020, and 2021. We used 3-month blocks of the annual 12-month calendar to sample our population in order to make our cohort as homogeneous as possible with regard to the incidence of certain pathologies with a seasonal trend, also considering the SARS-CoV-2 outbreak in 2020 and 2021. To exclude the possibility of SARS-CoV-2 infection, before admission, a double-negative PCR swab test for SARS-CoV-2 was required. Moreover, in patients with radiological and clinical elements indicating COVID-19, the research through PCR of SARS-CoV-2 on a sample from bronchus alveolar lavage was performed, according to hospital protocols. The collected data included: (1) age, gender, comorbidities (the presence of hypertension, chronic heart failure, diabetes mellitus, chronic kidney failure, neoplasm, chronic liver disease, previous stroke, and chronic obstructive pulmonary disease); (2) clinical events that had occurred during index hospitalization (death, days of in-hospital stay, diagnosis of sepsis, blood transfusion needed) consulting the hospital discharge form; (3) patient’s clinical and biochemical characteristics at the moment of hospitalization (systolic and diastolic blood pressure, glycemia, creatinine, estimated glomerular filtration rate (eGFR), total cholesterol, high-density lipoprotein cholesterol [HDL-c], triglycerides, low-density lipoprotein cholesterol [LDL-c], aspartate transaminase (AST), alanine transaminase (ALT), total proteins, albumin, *N*-terminal fragment brain natriuretic peptide (NT-proBNP), procalcitonin, complete blood count, hemoglobin, hematocrit, high sensitivity C-reactive protein (hs-CRP), and international normalized ratio (INR).

### 2.2. Lab Tests

Plasma glucose, total cholesterol, triglycerides, creatinine, AST, and ALT were measured with enzymatic assays, hs-CRP with immunoturbidimetric method, HDL-c with colorimetric Assay Kit (Double reagents) (Architect c System Abbot, Abbott Park, IL, USA). Blood count was assessed by impedancemetry, while the INR was calculated from prothrombin time, measured with a coagulative method.

### 2.3. Calculations

We assessed the CONUT score from the serum albumin level, TC concentration and peripheral lymphocyte count, in line with the results of the original study [10]. Albumin levels above 3.5 g/dL, between 3.0 and 3.49 g/dL, between 2.5 and 2.99 g/dL, and below 2.5 g/dL were given the scores 0, 2, 4, and 6, respectively. Scores of 0, 1, 2, and 3 were assigned to a lymphocyte count above 1600/mm^3^, between 1200 and 1599/mm^3^, between 800 and 1199/mm^3^ and below 800/mm^3^, respectively. TC levels above 180 mg/dL, between 140 and 179 mg/dL, between 100 and 139 mg/dL, and below 100 mg/dL were given the scores 0, 1, 2, and 3, respectively (Table 1). The CONUT score is the summation of the three scores. The following four groups of patients were defined based on the severity of undernutrition, as previously described [10]: normal (0–1), mild (2–4), moderate–high (5–8), and marked high (9–12). The formula from Chronic Kidney Disease Epidemiology Collaboration (CKD-EPI) was used to calculate eGFR [14]. The Padua Prediction Score for the risk of venous thromboembolism was calculated according to the original study [15]. We considered the high/low cut-off of NT-proBNP, procalcitonin, AST, ALT, and hs-CRP according to upper laboratory limits, as follows: NT-proBNP, 260 pg/mL; procalcitonin, 0.5 µg/L; AST, 34 UI/L; ALT, 55 UI/L; hs-CRP, 0.5 mg/dL.

### 2.4. Statistical Analysis

Data are presented as the mean SD or median (IQR). A Kolmogorov–Smirnov test was used to assess each variable’s distributional characteristics, including normality. An ANOVA for continuous variables and the Chi-square test for non-continuous variables were used for group comparisons. When necessary, numerical variables were logarithmically transformed to reduce skewness, and values are expressed as median and interquartile range. A *p*-value less than 0.05 was considered statistically significant. Statistical analysis was performed with Stat View 6.0 for Windows.

The primary end point of this study was the risk of all-cause death during the index hospitalization, according to the CONUT score. The secondary outcomes included the diagnosis of sepsis during hospitalization and days of in-hospital stays. To evaluate the risk of a high CONUT (5–12) relative to a low CONUT (0–4) score for the primary and secondary end points, we performed a multivariable logistic regression model. The model was adjusted for the following confounders: the presence of hypertension, chronic heart failure, type 2 diabetes mellitus, chronic kidney failure, neoplasm, previous stroke, chronic obstructive bronco-pneumopathy, and chronic liver disease.

## 3. Results

### 3.1. Baseline Characteristics, Medical History, and Comorbidities of the Patients According to the CONUT Score

**Figure 1:**
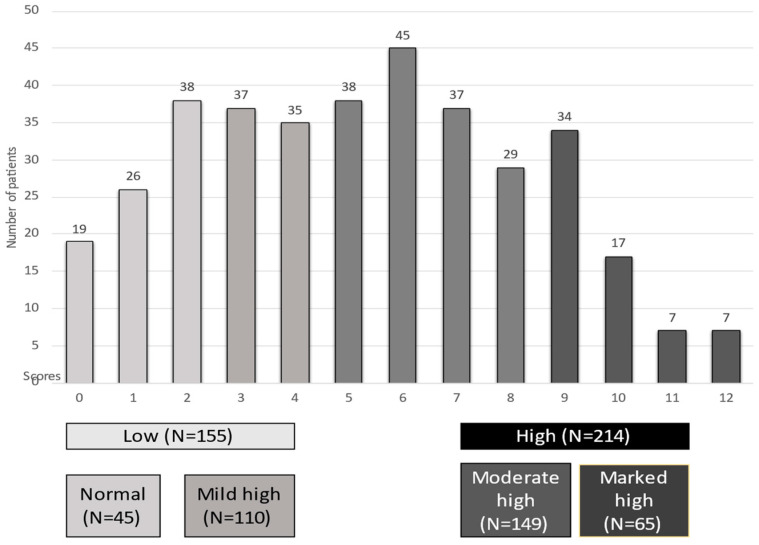
Distribution and classification of the study population according to the CONUT score.

Distribution and classification of the study population according to their CONUT score is shown in Figure 1. In total, data from 369 patients were retrospectively collected from medical records. The study population was divided into the following four groups based on their CONUT score: 45 patients with a normal score, 110 with a mild high score, 149 with moderate high score and 65 with a marked high score. Thus, the majority of patients had a high CONUT score (5–12) (*n* = 214), whereas 155 patients had a low CONUT score (0–4).

As concerns admission diagnoses, the most frequent reasons for admission to our department were infectious diseases, gastrointestinal, and neoplasms, with a percentage of 21% for both infectious and gastrointestinal diseases, and 14% for neoplasms during 2019. Moreover, we noticed that during the COVID pandemic, infectious diseases reached a percentage of 29%, gastrointestinal disease 20%, and 11% for neoplasms. Finally, during 2021, the percentage of infectious disease as a reason for hospitalization increased to 35%, whereas the percentage for gastrointestinal disease and neoplasms remained the same as 2020.

Clinical and biochemical characteristics of the study population according to their CONUT score are showed in Table 2 and Table 3. Patients in the moderate–high and marked high CONUT score groups were older (‘moderate–high’ vs. ‘normal’, *p* < 0.0001; ‘moderate–high’ vs. ‘mild’, *p* = 0.04; ‘marked high’ vs. ‘normal’, *p* < 0.0001; ‘marked high’ vs. ‘mild’ *p* = 0.004), more often male ( ‘moderate–high’ vs. ‘normal’, *p* = 0.01; ‘marked high’ vs. ‘normal’, *p* = 0.0004; ‘marked high’ vs. ‘mild’, *p* = 0.006), and were more likely to have a lower HDL-c (‘moderate–high’ vs. ‘normal’, *p* < 0.0001; ‘moderate–high’ vs. ‘mild’, *p* = 0.0006; ‘marked high’ vs. ‘normal’, vs. ‘mild’ and vs. ‘moderate–high’, *p* < 0.0001), total protein ( ‘moderate–high’ vs. ‘normal’ and vs. ‘mild’, *p* < 0.0001; ‘marked high’ vs. ‘normal’, vs. ‘mild’, and vs. ‘moderate high’ *p* < 0.0001), eGFR (‘moderate–high’ vs. ‘normal’, *p* < 0.0001; ‘moderate high’ vs. ‘mild’ *p* = 0.04; ‘marked high’ vs. ‘normal’, *p* = 0.001), and hemoglobin levels ( ‘moderate–high’ vs. ‘normal’, *p* < 0.0001; ‘moderate–high’ vs. ‘mild’ *p* = 0.002; ‘marked high’ vs. ‘normal’, vs. ‘mild’, and vs. ‘moderate–high’, *p* < 0.0001); in addition, they showed higher hs-CRP (‘moderate high’ vs. ‘normal’, *p* = 0.005; ‘marked high’ vs. ‘normal’, vs. ‘mild’, and vs. ‘moderate–high’, *p* < 0.0001) and the Padua Prediction Score (‘moderate high’ vs. ‘normal’ and vs. ‘mild’, *p* < 0.0001; ‘marked high’ vs. ‘normal’ and vs. ‘mild’, *p* < 0.0001). 

**Figure 2:**
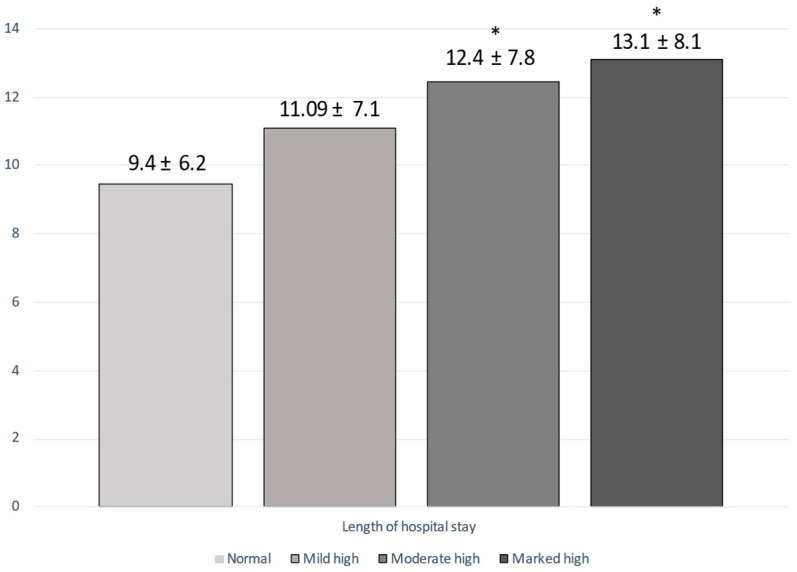
Difference in the length of hospital stay among the four groups, according to the CONUT score. * *p* < 0.05 vs. ‘normal’.

Medical history and comorbidities of the patients are showed in Table 4 and Table 5. Patients in the moderate–high and marked high CONUT groups were more likely to have type 2 diabetes mellitus (‘moderate–high’ vs. ‘normal’ *p* = 0.04; ‘marked high’ vs. ‘normal’, *p* = 0.002; ‘marked high’ vs. ‘mild’ *p* = 0.012), chronic kidney failure (‘moderate–high’ vs. ‘normal’ *p* = 0.002; ‘marked high’ vs. ‘normal’, *p* = 0.005), neoplasms (‘moderate–high’ vs. ‘mild’ *p* = 0.01; ‘marked high’ vs. ‘normal’ *p* = 0.01; ‘marked high’ vs. ‘mild’ *p* = 0.0008), COPD (‘moderate–high’ vs. ‘normal’, *p* = 0.01; ‘marked high’ vs. ‘normal’, *p* = 0.02), and blood transfusion needed during hospitalization (‘marked high’ vs. ‘normal’, *p* = 0.006; ‘marked high’ vs. ‘mild’ *p* = 0.03). The LOS was higher in the moderate–high and marked high CONUT score groups compared with other groups (‘moderate–high’ vs. ‘normal’, *p* = 0.02; ‘marked high’ vs. ‘normal *p* = 0.01) (Figure 2).

### 3.2. Clinical Outcomes According to the CONUT Score Groups

**Figure 3:**
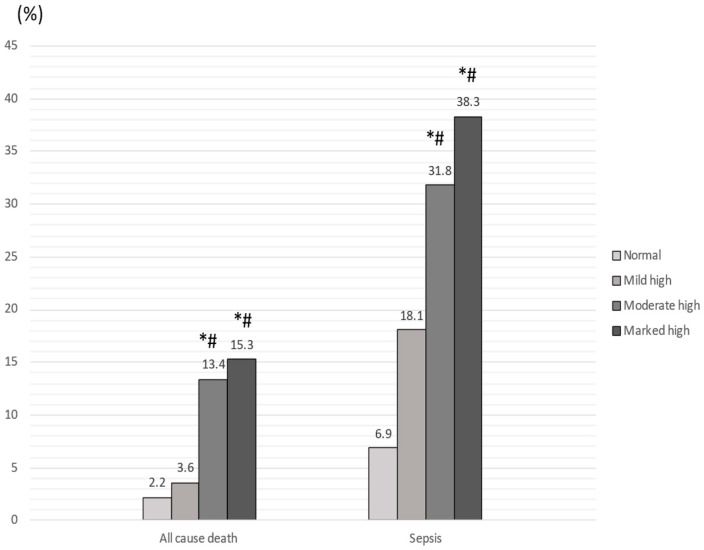
Distribution of all-cause death and sepsis among the four groups according to the CONUT score. * *p* < 0.05 vs. ‘normal’; # *p* < 0.05 vs. ‘mild’.

The primary end point (in hospital all-cause mortality) occurred in one patient (2.2%) in the normal CONUT score group, four patients (3.6%) in the mild CONUT score group, 20 patients (13.4%; vs. ‘normal’, *p* = 0.002; vs. ‘mild’, *p* = 0.007) in the moderate–high CONUT score group, and 10 patients (15.3%; vs. ‘normal’, *p* = 0.02; vs. ‘mild’, *p* = 0.01) in the marked high CONUT score group (Figure 3). In regards to secondary outcomes, diagnosis of sepsis occurred in 72 patients (33.8%) in the high CONUT score group vs. 23 patients (14.9%) in low CONUT score group (*p* < 0.0001) (Figure 3), while hospitalization longer > 12 days occurred in 103 patients (48.1%) in the high CONUT score group vs. 48 patients (30.9%) in the low CONUT score group (*p* = 0.001)

### 3.3. Multivariable Logistic Analysis and Subgroup Analysis for the Primary Outcome Measure; High versus Low CONUT Score Groups

Multivariable logistic analysis was performed and our patients were distributed into two groups: those with a CONUT score of 5–12 (high CONUT) and those with a CONUT score of 0–4 (low CONUT) in order to evaluate the risk of a high CONUT relative to a low CONUT for the primary and secondary end points. After adjusting for confounders, the excess risk of a high CONUT score (5–12) relative to a low CONUT score (0–4) for in-hospital all-cause death remained significant (OR 3.3, 95% CI (1.1–9.7), *p* = 0.02).

Even after adjusting for confounders, the excess risk of a high CONUT score relative to a low CONUT score group for diagnosis of sepsis and hospitalization > 12 days remained statistically significant (OR 2.7, CI (1.5–4.9), *p* = 0.01 and OR 2.1, CI (1.2–3.9), *p* = 0.007 respectively).

**Figure 4:**
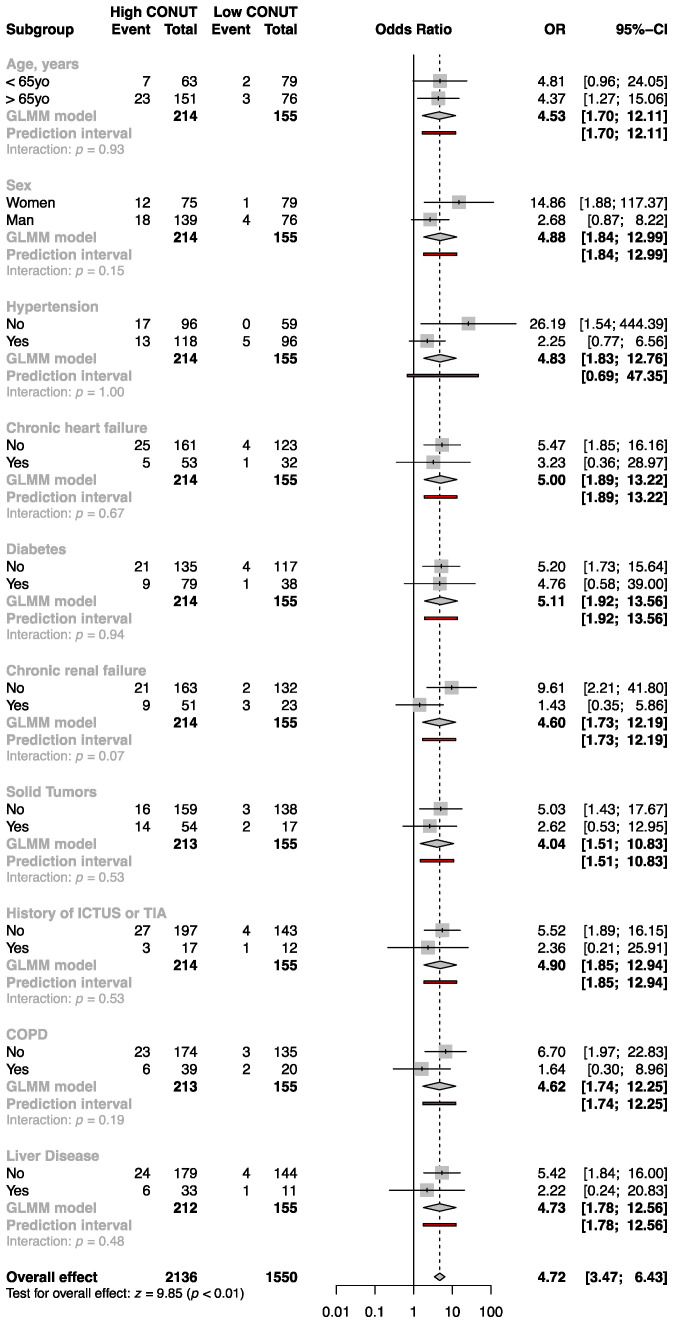
Subgroup analysis for the primary outcome measure: high versus low CONUT score groups.

In the subgroup analysis, the study population was stratified by age, sex, presence of hypertension, chronic heart failure, type 2 diabetes, chronic renal failure, solid tumors, history of stroke, COPD, and liver disease, there was no significant interaction between the subgroup variables and the result of a high CONUT score relative to a low CONUT score for in-hospital all-cause mortality (Figure 4).

## 4. Discussion

This study examined the CONUT score as a prognostic tool to predict in-hospital death, infections, and LOS in adult patients admitted in a department of internal medicine. The principal findings were: (1) patients with a high CONUT score at admission had an increased risk of in-hospital mortality, even after correction for covariates; (2) the adjusted additional risk of sepsis during hospitalization, as well as the risk of a longer LOS, were significantly increased among those with a high CONUT score (5–12). Malnutrition is quite common among patients admitted to internal medicine departments, and the majority of these patients require urgent multidisciplinary management [16]. Accordingly, the identification of a simple score with a high predictive value is mandatory to properly address patients’ nutritional needs. Our findings indicated that consideration should be given to the CONUT score for nutritional assessment of patients hospitalized in an internal medicine ward. The CONUT score has indeed been shown to be helpful in identifying hospitalized patients with malnutrition and poor clinical outcomes; however, prior studies have focused on different clinical scenarios, such as oncology and cardiology. The association between malnutrition, as measured by the CONUT score and overall mortality in patients with heart failure, was underlined by two studies [17,18]. In addition, these results were confirmed in another study: the MNA was recommended by Hu et al. as the nutritional tool for patients with heart failure; however, they also highlighted the CONUT score as a predictor of overall mortality [19]. In the context of oncology, two recent metanalyses showed that a high CONUT score is associated with poor overall survival in patients with renal cell carcinoma, urothelial carcinoma, and pancreatic cancer [20,21]; furthermore, Peng et al. demonstrated that a high CONUT score correlates with worse prognoses in patients with non-small cell lung cancer [22]. In this study, we found a higher risk of diagnosis of sepsis in subjects with a higher CONUT score. Similar results were previously reported by other authors: in a study examining the risk of in-hospital mortality and infections in patients with acute heart failure, Kato et al. found that a high CONUT score was related to an excess risk of infection [17]. Additionally, Qian et al. found that patients undergoing surgical gastrectomy who had a CONUT score >2 had a greater rate of infections [23]. The CONUT score is a comprehensive tool that easily evaluates malnutrition using routinary blood biochemical tests for hospitalized patients. It displays the immunological and inflammatory condition, as well as the storage of proteins and lipids. All the elements of the CONUT score have been linked to a worse prognosis among patients. Particularly, hospitalized individuals with low serum albumin have been found to have an elevated death rate [20,24,25]. Thus, albumin blood concentrations could be considered a predictor of in-hospital complications and mortality. A possible explanation is that hypoalbuminemia leads to oxidative stress with cellular damage and apoptosis. Together with albumin dosage, a low TC and lymphocyte count are also associated with higher mortality, reflecting a deterioration in nutritional status and a decreased immune and inflammatory status [26,27]. The CONUT score carried out at the moment of admission and represented a vicious cycle well; indeed, patients with multimorbidity tend to suffer malnutrition through many mechanisms, such as fluid retention, long bed stay, lack of appetite leading to inflammation, and neurohormonal activation [28]. Our findings are consistent with previous studies. Indeed the CONUT score was effective in differentiating inpatients with and without adverse outcomes for a variety of diseases, as described above.

The present study has several strengths. To the best of our knowledge, this is the first study to investigate the prognostic value of the CONUT score in internal medicine ward patients in the short term. Indeed, the predictive value of a high CONUT score at the moment of admission may enable clinicians to recognize patients at risk for adverse outcomes, who may benefit from nutritional supplementation. However, several limitations should be highlighted. First, this is a retrospective study from an internal medicine department with limited sample sizes, and this limits the statistical power of the study. Second, data regarding statin use, which can affect the CONUT score via influencing cholesterol dosage, were not collected from our patients. Third, we conducted large statistical adjustment for the measured confounders, however, there are unmeasured variables affecting the in-hospital prognosis that may not have been taken into consideration, such as administered drugs, adverse reactions, and nosocomial infections. Additionally, even though some patients received nutritional and counselling services from the Nutrition Support Team during hospitalization, there is no information available about their nutritional status or compliance after discharge, so we are unable to draw any conclusions about their long-term prognosis (i.e., re-hospitalization rate, death during the first month, loss of autonomy in activity of daily living, etc.). Longitudinal studies, with a follow-up after hospital discharge, are needed to evaluate this aspect.

## 5. Conclusions

The CONUT score shows high prognostic value for in-hospital mortality, risk of sepsis, and a longer LOS in the clinical setting of internal medicine departments. These data suggest a possible use of the CONUT score as a nutritional screening tool in identifying those patients with higher risk of adverse in-hospital outcomes. The predictive role of different CONUT score cut-off values needs to be validated in populations with different diseases in future multicenter, large-sample, prospective clinical studies.

## Figures and Tables

**Table 1:** Score categories for the CONUT score.

Lymphocyte (/mL)	Points	Total Cholesterol (mg/dL)	Points	Albumin (g/dL)	Points
<800	3	<100	3	<2.5	6
800–1200	2	100–140	2	2.5–3	4
1200–1600	1	140–180	1	3–3.5	2
>1600	0	>180	0	>3.5	0

**Table 2:** Clinical and biochemical characteristics of the patients according to normal (0–2), mild (3–4), moderate–high (5–8), or marked high (9–12) CONUT Score.

	CONUT Score			
	Low		High	
	Normal (0–2) *n* = 45	Mild (3–4) *n* = 110	Moderate–High (5–8) *n* = 149	Marked High (9–12) *n* = 65
Age (years)	56.6 ± 21.1	64.6 ± 19.7 *	69.1 ± 16.1 *#	72.3 ± 9.7 *#
Sex (M%)	40	52.7	61 *	73.8 *#
Systolic Pressure (mmHg)	125.3 ± 18.8	126.2 ± 17.6	126.5 ± 20.5	123.2 ± 20.4
Diastolic Pressure (mmHg)	70.3 ± 10.9	74.0 ± 12.3	71.6 ± 9.7	69.2 ± 10.5 #
Glycemia (mg/dL)	95.9 ± 36.4	112.6 ± 56.6	110.1 ± 47.7	112.7 ± 57.2
Serum Creatinine (mg/dL)	0.7 ± 0.3	1.3 ± 1.8 *	1.3 ± 1.3 *	1.1 ± 1.2
eGFR (ml/min/1.74 m^2^)	94.7 ± 27.5	78.8 ± 33.7 *	70.7 ± 34.4 *#	73.8 ± 29.1 *
Total cholesterol (mg/dL)	189.4 ± 35.9	164.4 ± 48.5 *	137.2 ± 40.2 *#	102.4 ± 26.3 *#@
Triglycerides (mg/dL)	134.7 ± 56.7	128.9 ± 65.1	130.2 ± 62.4	103.4 ± 44.5
HDL-c (mg/dL)	46.5 ± 13.7	37.5 ± 14.6 *	31.6 ± 13.6 *#	21.2 ± 9.1 *#@
LDL-c (mg/dL)	115.8 ± 34.7	100.2 ± 43.8 *	80.6 ± 35.3 *#	60.3 ± 23.6 *#@
Total Proteins (g/dL)	6.8 ± 0.5	6.3 ± 0.6 *	5.8 ± 0.6 *#	5.3 ± 0.7 *#@
Albumin (g/dL)	4.5 ± 4.0	3.5 ± 0.4 *	2.9 ± 0.4 *#	2.4 ± 0.3 *#@
AST (U/L) ≥ 35 (%)	24	25	30	37
ALT (U/L) ≥ 56 (%)	18	21	17	12
NT-proBNP > 260 (pg/mL) (%)	4.4	23.6 *	22.1 *	30.7 *
Procalcitonin > 0.5 (µg/L) (%)	2.2	13.6 *	27.5 *#	30.7 *#
Hs-CRP (mg/dL)	2.4 ± 3.4	6.1 ± 8.8	8.1 ± 8.1 *	16.9 ± 22.2 *#@
Padua Prediction Score	2.2 ± 1.8	2.9 ± 1.8 *	3.8 ± 1.7 *#	4 ± 1.6 *#
WBC (10^3^/μL)	9.6 ± 6.2	8.9 ± 4.6	11.0 ± 14.7	8.0 ± 5.9
Lymphocyte (10^3^/μL)	1.8 (1.6–2.8)	1.6(1.17–2) *	1.2(0.87–1.7) *	0.7 (0.5–1.1) *
RBC (10^6^/μL)	4.4 ± 0.5	3.9 ± 0.8	6.5 ± 30.1	3.5 ± 0.8
Hemoglobin (g/dL)	13.3 ± 4.6	11.4 ± 2.2 *	10.4 ± 1.8 *#	9.7 ± 1.8 *#@
Hematocrit (%)	38.7 ± 9.5	34.3 ± 6.2 *	31.4 ± 5.6 *#	30.4 ± 8.1 *#
PLT (10^3^/μL)	253 ± 94	243 ± 142	222 ± 121	188 ± 122 *#
INR	1.9 ± 4.4	2.1 ± 5.2	1.6 ± 4.0	1.3 ± 0.2

Data are presented as the mean SD, median (IQR) or percentage (%). HDL-c, HDL cholesterol; LDL-c, LDL cholesterol; eGFR, estimated glomerular filtration rate; AST, aspartate transaminase; ALT, alanine transaminase; NT-proBNP, *N*-terminal fragment brain natriuretic peptide; hs-CRP, high sensitivity C-reactive protein; WBC, white blood cells; RBC, red blood cells, PLT, platelets; INR, international normalized ratio. * *p* < 0.05 vs. ‘normal’; # *p* < 0.05 vs. ‘mild’; @ *p* < 0.05 vs. moderate–high.

**Table 3:** Clinical and biochemical characteristics of the patients according low (0–4) or high (5–12) CONUT Score.

	CONUT Score	
	Low (0–4) (*n* = 155)	High (5–12) (*n* = 214)
Age (years)	62.3 ± 20.4	70.1 ± 14.6
Sex (M%)	49	65 *
Systolic Pressure (mmHg)	125.9 ± 17.9	125.5 ± 20.6
Diastolic Pressure (mmHg)	72.9 ± 12.0	70.9 ± 10.0
Glycemia (mg/dL)	107.8 ± 52.1	110.9 ± 50.6
Serum Creatinine (mg/dL)	1.18 ± 1.6	1.27 ± 1.33
eGFR (mL/min/1.74 m^2^)	83.4 ± 32.8	71.6 ± 32.9
Total cholesterol (mg/dL)	171.8 ± 46.5	127.1 ± 39.9 *
Triglycerides (mg/dL)	130.6 ± 62.7	122.4 ± 58.9
HDL-c(mg/dL)	40.2 ± 14.9	28.6 ± 13.4 *
LDL-c (mg/dL)	104.8 ± 41.9	74.7 ± 33.6 *
Total Proteins (g/dL)	6.5 ± 0.7	5.7 ± 0.7 *
Albumin (g/dL)	3.8 ± 2.2	2.7 ± 0.4 *
AST (U/L) ≥ 35 (%)	25	32
ALT (U/L) ≥ 56 (%)	20	15
NT-proBNP > 260 (pg/mL) (%)	18	24.7
Procalcitonin > 0.5 (µg/L) (%)	10.3	28 *
Hs-CRP (mg/dL)	5.0 ± 7.8	10.7 ± 14.4 *
Padua Prediction Score	2.7 ± 1.8	3.9 ± 1.7 *
WBC (10^3^/μL)	9.1 ± 5.1	10.0 ± 12.6
Lymphocyte (10^3^/μL)	1.7 (1.3–2.2)	1.0 (0.7–1.5) *
RBC (10^6^/μL)	4.1 ± 0.7	3.6 ± 0.8 *
Hemoglobin (g/dL)	11.9 ± 3.2	10.2 ± 1.8 *
Hematocrit (%)	35.6 ± 7.6	31.1 ± 6.5 *
PLT (10^3^/μL)	247 ± 130	211 ± 122 * 1.56 ± 3.3
INR	2.1 ± 4.9	

Data are presented as the mean SD, median (IQR) or percentage (%); HDL-c, HDL cholesterol; LDL-c, LDL cholesterol; eGFR, estimated glomerular filtration rate; AST, aspartate transaminase; ALT, Alanine transaminase; NT-proBNP, *N*-terminal fragment brain natriuretic peptide; hs-CRP, high sensitivity C-reactive protein; WBC, white blood cells; RBC, red blood cells; PLT, platelets; INR, international normalized ratio. * *p* < 0.05 vs. ‘low’.

**Table 4:** Medical history and comorbidities of the patients according to normal (0–2), mild (3–4), moderate–high (5–8), and marked high (9–12) CONUT score.

	CONUT Score			
	Low		High	
	Normal (0–2) *n* = 45	Mild (3–4) *n* = 110	Moderate–High (5–8) *n* = 149	Marked High (9–12) *n* = 65
Blood Transfusion (%)	8.8	16.3	18.8	29.7 *#
Diabetes Mellitus (%)	17.7	27.2	34.2 *	45.1 *#
Hypertension (%)	48.8	67.2 *	61	44.2 #@
Chronic Heart Failure (%)	13.3	23.6	24.8	26.2
Previous Stroke (%)	6	13.5	10	12.2
Chronic Kidney Failure (%)	2.2	19.1 *	24.1 *	8.3 *
Neoplasm (%)	18.1	14.8	30.5 #	39.1 *#
Chronic Obstructive Pulmonary Disease (%)	6.7	17.2	24.1 *	25 *
Chronic Liver Disease (%)	6.6	7.2	11.4	26.2 *#@

Data are presented as the percentage (%). * *p* < 0.05 vs. ‘normal’; # *p* < 0.05 vs. ‘mild’; @ *p* < 0.05 vs. moderate–high.

**Table 5:** Medical history and comorbidities of the patients according to low (0–4) or high (5–12) CONUT score.

	CONUT Score	
	Low (0–4) *n* = 155	High (5–12) *n* = 214
Blood Transfusion (%)	14.2	22.0 *
Diabetes Mellitus (%)	24.5	32.7 *
Hypertension (%)	61.9	51.4
Chronic Heart Failure (%)	20.6	25.2
Previous Stroke (%)	11.6	10.7
Chronic Kidney Failure (%)	14.2	19.1 *
Neoplasm (%)	15.6	32.7 *
Chronic Obstructive Pulmonary Disease (%)	14.1	24.2 *
Chronic Liver Disease (%)	7.1	15.9 *

Data are presented as percentage (%). * *p* < 0.05 vs. ‘low’.

## Data Availability

Data for this study are available upon request.
